# Foxp3+ Regulatory T Cells Inhibit CCl_4_-Induced Liver Inflammation and Fibrosis by Regulating Tissue Cellular Immunity

**DOI:** 10.3389/fimmu.2020.584048

**Published:** 2020-10-15

**Authors:** Yoshinobu Ikeno, Daiya Ohara, Yusuke Takeuchi, Hitomi Watanabe, Gen Kondoh, Kojiro Taura, Shinji Uemoto, Keiji Hirota

**Affiliations:** ^1^Department of Hepatobiliary, Pancreas and Transplant Surgery, Graduate School of Medicine, Kyoto University, Kyoto, Japan; ^2^Laboratory of Integrative Biological Science, Institute for Frontier Life and Medical Sciences, Kyoto University, Kyoto, Japan

**Keywords:** liver fibrosis, regulatory T cells, Foxp3, amphiregulin, liver inflammation

## Abstract

Foxp3+ regulatory T (Treg) cells are pivotal in maintaining immunological self-tolerance and tissue homeostasis; however, it remains unclear how tissue Treg cells respond to liver injury and regulate chronic inflammation, which can cause liver fibrosis. We report here that hepatic Treg cells play a critical role in preventing liver pathology by suppressing inflammatory cellular immunity that can promote liver damage and fibrosis. Chronic liver inflammation induced by injections of carbon tetrachloride (CCl_4_) led to preferential expansion of hepatic Treg cells that prevented liver fibrosis. In contrast, depletion of Treg cells in the CCl_4_-induced liver fibrosis model exacerbated the severity of liver pathology. Treg depletion unleashed tissue cellular immunity and drove the activation and expansion of the pro-fibrotic IL-4-producing T helper 2 cells, as well as CCR2^high^ Ly-6C^high^ inflammatory monocytes/macrophages in the inflamed liver. Although Treg expression of amphiregulin plays a key role in tissue remodeling and repair in various inflammation models, amphiregulin from hepatic Treg cells, the largest producer among liver immune cells, was dispensable for maintaining liver homeostasis and preventing liver fibrosis during CCl_4_-induced chronic inflammation. Our results indicate that Treg cells control chronic liver inflammation and fibrosis by regulating the aberrant activation and functions of immune effector cells. Harnessing Treg functions, which effectively regulate tissue cellular immunity, may be a therapeutic strategy for preventing and treating liver fibrosis.

## Introduction

Liver fibrosis is characterized by the excessive accumulation of extracellular matrix proteins such as collagen, resulting from chronic liver inflammation mediated by infectious agents, excessive alcohol abuse, non-alcoholic steatohepatitis (NASH), and autoimmune liver diseases (1). It is widely appreciated that various types of inflammatory immune cells mediate chronic tissue inflammation in response to liver injury and the pathogenesis of liver fibrosis. For example, abundant inflammatory monocytes and macrophages are recruited to the inflamed liver tissue and are involved in the augmentation of liver fibrosis. The imbalance of IFN-γ-producing T helper (Th1) cells and IL-4-producing T helper (Th2) cells, which are the anti-fibrotic and pro-fibrotic Th subsets, respectively, also promotes liver fibrosis (2). Thus, activated innate and adaptive immune cells stimulate hepatic stellate cells to secrete extracellular matrix proteins, resulting in fibrotic changes in the liver that severely impair physiological liver functions. However, the mechanisms by which fibrotic tissue immune responses in the inflamed liver are negatively regulated remain unknown.

Foxp3+ regulatory T (Treg) cells play a pivotal role in maintaining immunological self-tolerance and tissue homeostasis by regulating inappropriate immune responses to self-organs and tissues (3). Under various pathogenic conditions in humans and animal models, Treg cells are recruited into the liver to regulate tissue inflammation and pathology (4–8). There is also accumulated evidence that tissue Treg cells actively engage in tissue remodeling and repair in stressed or damaged organs, for example, by secreting amphiregulin, a key molecule for tissue regeneration and repair in various inflammation models, including brain ischemia, muscle injury, and viral pneumonia (9–11). Thus, it is becoming increasingly clear that Treg cells have tissue-specific transcriptional signatures and functions to control tissue injury in a context-dependent manner. However, little is known about the mode of Treg actions regulating chronic liver inflammation and fibrosis.

Here, using a carbon tetrachloride (CCl_4_)-induced liver fibrosis model, we investigated the dynamics of cellular and molecular signatures in the inflamed liver regulated by Treg cells and Treg-specific amphiregulin.

## Materials and Methods

### Mice

C57BL/6J mice were purchased from CLEA Japan. Foxp3-DTR-eGFP (FDG) mice (12) and Foxp3^YFP–Cre^ mice (13) were purchased from the Jackson Laboratory. Areg^fl/fl^ mice were generated by inserting Loxp3 sites flanking exons 3 and 4 of the murine *Areg* gene using the CRISPR/Cas9 system. These mice were crossed with Foxp3^YFP–Cre^ mice. All mice used had a C57BL/6J background and were maintained under specific-pathogen-free conditions. All animal experiments were approved by the Ethical Committee of Institute for Frontier Life and Medical Sciences and Graduate School of Medicine, Kyoto University. All relevant experiments were performed in accordance with the institutional guidelines.

### Mouse Models of Liver Fibrosis and *in vivo* Depletion of Treg Cells

Liver fibrosis was induced by intraperitoneal (i.p.) injections of CCl_4_ (0.5 μl/g body weight; Wako) diluted 1:4 in corn oil (Wako) twice a week for 6 weeks as described previously (14). In brief, CCl_4_ is converted into several radical forms by liver cytochrome P450 enzymes. The resulting radicals induce hepatotoxic damage and result in fibrosis. Mice were sacrificed 3 days after the final CCl_4_ injection. Liver fibrosis was also induced by feeding choline-deficient L-amino acid-defined (CDAA) diet for 8 weeks (Dyets Inc, Bethlehem, PA) (15). In brief, CDAA diet affects lipid metabolisms, resulting in steatohepatitis and liver fibrosis (16). For Treg-cell depletion experiments, FDG mice were injected i.p. with diphtheria toxin (DT; 1 μg in 100 μl PBS; Merck) every other day for 2 weeks (Figure 2A and Supplementary Figure 1A).

### Alanine Transaminase Measurement and Liver Histology

Serum alanine transaminase (ALT) levels were measured using an Infinity kit (Thermo Fisher Scientific) in accordance with the manufacturer’s instructions. Formalin-fixed frozen livers were stained with hematoxylin-eosin (Wako) or Sirius red (Wako) for histological analysis. The regions of the entire median lobe of the liver stained with Sirius red were extracted and quantified using Image J software (NIH). The mean percentage of the Sirius red-positive area was determined from four images of each mouse.

### Preparation of Single-Cell Suspension From the Liver and Flow Cytometry

The collected livers were cut into small pieces and mashed through a 70 μm mesh filter. Leukocytes were then enriched by 37.5% Percoll (GE Healthcare) separation, followed by red blood cell lysis with RBC lysis buffer (Sigma-Aldrich). The resulting single-cell suspensions were incubated with anti-mouse CD16/32 antibody (2.4G2; BD Bioscience) and Fixable Viability Dye eFluor^TM^ 780 (eBioscience). The cells were then stained using fluorescent conjugated antibodies against cell surface markers. For Foxp3 and amphiregulin staining, the Foxp3/Transcription Factor Staining Buffer Set (eBioscience) was used in accordance with the manufacturer’s instructions. For intracellular cytokine staining, cells were re-stimulated with phorbol12-myristate 13 acetate (50 ng/ml; Sigma-Aldrich) and ionomycin (500 ng/ml; Sigma-Aldrich) in the presence of Brefeldin A (1 μg/ml; Merck) in IMDM (Sigma-Aldrich) containing 5% FBS (GIBCO), penicillin-streptomycin (Nacalai Tesque), 2-mercaptoethanol (GIBCO), GlutaMAX (GIBCO), sodium pyruvate (GIBCO), and MEM NEAA (GIBCO) for 4 h. Each sample was then fixed using 3.7% formaldehyde (Sigma-Aldrich), permeabilized with 0.1% NP-40 (Nacalai Tesque), and stained with antibodies against cytokines. The monoclonal antibodies used for flow cytometry analysis were as follows: anti-mouse CD3e (145-2C11), CD25 (PC61), Ly-6C (AL-21), IL-4 (11B11), Ki-67 (B56), PE-Streptavidin (all from BD Bioscience), anti-mouse CD4 (RM4-4), CD8a (53-6.7), CD11b (M1/70), CD45.2 (104), F4/80 (BM8), IL-33Ra (D1H9), Ly-6G (1A8), CCR2 (SA203G11), IL-17A (TC11-18H10.1) (all from Biolegend), anti-mouse Foxp3 (FJK-16s), and IFN-γ (XMG1.2) (both from eBioscience), and anti-mouse amphiregulin-biotin (BAF989; R&D Systems). All flow cytometry data were acquired using a BD FACSCantoII or BD FACS AriaSORP devise and analyzed using FlowJo software (Tree Star, Inc.).

### IL-33 Measurement in the Liver Tissue

Liver tissues were homogenized in PBS containing a cocktail of protease inhibitors using MagNALyzer (Roche). After centrifugation, IL-33 in the supernatant was measured using Mouse IL-33 ELISA Kit (Biolegend). The data was normalized by the original liver weight.

### Quantitative RT-PCR

For extraction of total RNA from hepatic immune subsets, cells were directly sorted into 500 μl of TRIzol (Invitrogen) and were lysed by vortexing. For extraction of total RNA from liver tissues, the tissue was homogenized in 500 μl of Trizol using MagNALyzer. After RNA extraction, genomic DNA was removed using an RNeasy mini kit and RNase-Free DNase Set (QIAGEN). Purified RNA was reverse-transcribed with SuperScript VILO (Invitrogen) and the cDNA was used for quantitative PCR with Fast SYBR Green Master Mix (Applied Biosystems) on a LightCycler 480 (Roche). The expression levels of target genes were quantified after normalization to 18S rRNA expression. The sequence primers used for quantitative RT-PCR were as follows: aSMA-F: 5′CTGGGACGACATGGAAAAG3′, aSMA-R: 5′GTT CAGTGGTGCCTCTGTCA3′, Col1a1-F: 5′ACATGTTCAGCT TTGTGGACC3′, Col1a1-R: 5′TAGGCCATTGTGTATGCAGC3′, Areg-F: 5′GACTCACAGCGAGGATGACA3′, Areg-R: 5′GGCT TGGCAATGATTCAACT3′, 18s-F: 5′AGTCCCTGCCCTTTGT ACACA3′, and 18s-R: 5′CGATCCGAGGGCCTCACTA3′.

### Library Preparation and RNA Sequencing Analysis

DNase-treated total RNA (1 μg) from liver tissues was used to construct a sequencing library using the NEBNext Poly(A) mRNA Magnetic Isolation Module (NEB) and NEBNext Ultra II RNA Library Prep Kit for Illumina (NEB) in accordance with the manufacturer’s instructions. The library DNA was sequenced using NextSeq 500 (Illumina). The sequenced reads were aligned to the UCSC mm10 mouse genome using Hisat2 (17) (version 2.1.0). The read counts of each gene were obtained using featureCounts (18) (version 1.6.4). Log2 fold change and adjusted *p* value of differential gene expression were calculated and statistically evaluated using DESeq2 (19) (version 1.20.0). Transcript per million (TPM) was calculated from read counts data and then each gene expression is normalized to Log10(TPM + 1). In Figure 4E, the expression of marker genes of inflammatory monocytes and macrophages are shown. For gene set enrichment analysis (GSEA) (Figure 4F), microarray gene expression datasets (E-MEXP-3177) (20) were analyzed to generate gene signatures of hepatic myeloid cell subsets using the limma package (21) (version 3.38.3) in R (version 3.5.1). Genes with an adjusted *p* value < 0.05 were ranked by log2 fold change. The top 200 up- and down-regulated genes were defined as hepatic Ly-6C^high^ inflammatory monocyte/macrophage and Ly-6C^low^ macrophage gene signatures, respectively, and used to make gene set files. Then, ranked list files for GSEA were generated by using log2 fold change from DESeq2 analysis of CCl_4_-treated Treg-depleted DT(+) and DT(−) livers. These data files were processed with GSEA v3.0 software (22) and the resultant enrichment plots were shown.

### Statistical Analysis

Statistical analyses were performed using GraphPad PRISM7 (GraphPad Software). A two-tailed *t*-test or ANOVA followed by Tukey’s multiple comparisons test was used for statistical hypothesis testing. A *p*-value < 0.05 was considered statistically significant. Bar graphs are presented as mean ± standard deviation (SD). The sample sizes for all data are described in the figure legends.

## Results

### Hepatic Treg Cells Proliferate and Suppress Liver Fibrosis Progression During CCl_4_-Induced Chronic Liver Inflammation

**FIGURE 1:**
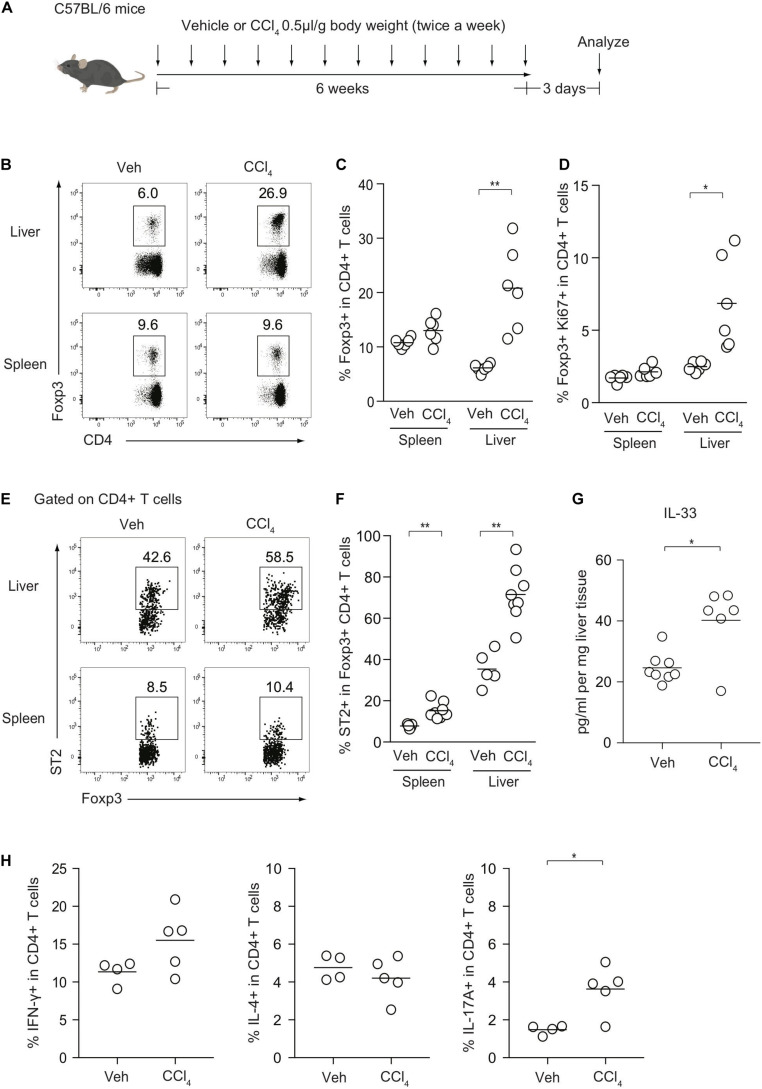
Kinetics of Foxp3+ Treg cells and effector Th subsets after CCl_4_-induced chronic liver injury. **(A)** Experimental design of CCl_4_-induced liver injury. **(B)** Flow cytometry analysis of hepatic and splenic CD4+ T cells for Foxp3 expression after intraperitoneal injections of either CCl_4_ or vehicle (Veh) as illustrated in panel **(A)**. **(C,D)** Proportions of Foxp3+ or Ki67+ Foxp3+ in CD4+ T cells in the spleen or liver (*n* = 6). **(E,F)** Proportions of ST2+ in Foxp3+CD4+ Treg cells in the spleen or liver (*n* = 5–8). **(G)** Quantification of IL-33 protein by ELISA in CCl_4_- or vehicle-treated livers (*n* = 6–8). **(H)** Proportions of IFN-γ, IL-4, and IL-17-producing CD4+ T cells in the liver from CCl_4_- (*n* = 5) or Veh-treated mice (*n* = 4). Horizontal bars indicate the means in panels **(C,D,F–H)**; *p < 0.05, **p < 0.01. Data are representative of three independent experiments in panels **(B,C)**, and two independent experiments in panels **(E–G)** and pooled from two independent experiments in panels **(D,H)**.

To investigate the cellular dynamics of CD4+ T cells during liver inflammation, we induced chronic liver injury and fibrosis in an established procedure by injecting CCl_4_ for 6 weeks (Figure 1A). The kinetics of Foxp3+ Treg cells and effector Th subsets were then analyzed. The frequencies of Foxp3^+^ Treg cells were significantly increased in the livers of CCl_4_-treated mice, compared with those in the livers of vehicle-treated control mice, while the levels of Treg cells in the spleen were comparable between the two groups (Figures 1B,C). Consistently, the fraction of Ki67+ Treg cells from the CCl_4_-treated liver was significantly increased (Figure 1D). Notably, ST2 expression of hepatic Treg cells was higher than splenic Treg cells and after CCl_4_ injections, the frequencies of ST2+ Treg cells were significantly increased and correlated with protein levels of IL-33, suggesting that Treg expansion may be driven by IL-33 released from damaged hepatocytes (Figures 1E–G). In terms of effector Th subsets, IL-17A-producing T helper (Th17) cells were slightly increased in the CCl_4_-treated liver, whereas the proportions of Th1 and Th2 cells in the liver between the groups were unchanged (Figure 1H).

**FIGURE 2:**
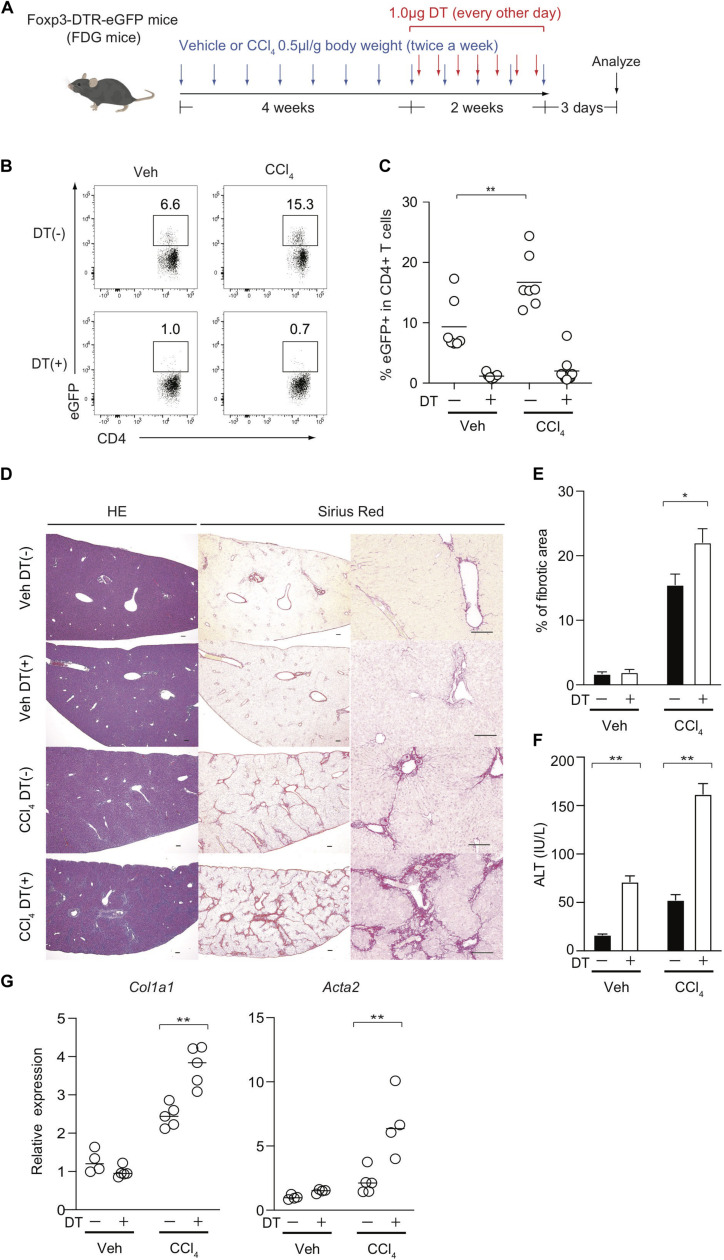
Treg-cell depletion aggravates CCl_4_-induced liver damage and fibrosis. **(A)** Experimental design of Treg-cell depletion during CCl_4_-induced liver injury. FDG mice were untreated or administered diphtheria toxin (DT) during the late phase of CCl_4_ and vehicle (Veh) treatment. **(B,C)** Proportions of eGFP+ in hepatic CD4+ T cells of FDG mice with DT(+) or without DT(−) as in panel **(A)**. **(D)** Representative images of hematoxylin and eosin (H&E) and Sirius red (SR) staining of the liver of DT(−) and DT(+) FDG mice. Scale bars indicate 100 μm. **(E)** Quantification of fibrotic areas of the liver section evaluated by SR-positive area (*n* > 5). **(F)** Serum level of ALT of DT(−) and DT(+) FDG mice (*n* > 5). Vertical bars denote SD. Horizontal bars indicate the means in pannels **(C,G)**. **(G)** Quantitative RT-PCR analysis for the expression of *Col1a1* and *Acta2* (α-SMA) in the liver tissue of DT(−) and DT(+) FDG mice (*n* = 4). Horizontal bars indicate the means; *p < 0.05, **p < 0.01. Data are representative of two independent experiments in panel **(B)** and are pooled in panel **(C)**. Data are representative of three independent experiments in panels **(E–G)**.

The observation of the preferential Treg expansion in damaged livers prompted us to examine the role of Foxp3^+^ Treg cells in the regulation of CCl_4_-induced liver inflammation and fibrosis. To this end, we removed Treg cells in the course of the development of the model using Foxp3-DTR-eGFP knock-in (FDG) mice in which Foxp3+ Treg cells are labeled as eGFP+ cells and can be specifically depleted by the administration of DT (Figure 2A). We confirmed that hepatic Treg cells in FDG mice were successfully depleted in both groups (Figures 2B,C). Using this system, we next evaluated if Treg depletion influenced the fibrotic regions of the liver as assessed by Sirius red staining and the serum levels of ALT, a marker of hepatocyte damage. Notably, the absence of Treg cells for the last 2 weeks over the course of CCl_4_ injections significantly increased the area of collagen deposition and the amounts of ALT in the sera compared with DT-untreated mice (Figures 2D–F). Although liver injury based on serum ALT levels could be exacerbated by Treg depletion alone, this acute inflammation for a limited period was not sufficient to induce liver fibrosis and the severe fibrosis required a combination of chronic inflammation by CCl_4_ and leukocyte infiltration in the liver by Treg ablation (Figures 2D–F). Consistent with collagen deposition in histology, the expression levels of the *Col1al* and *Acta2* (α-SMA) markers for tissue fibrosis were significantly increased in the inflamed liver of CCl_4_- and DT-injected FDG mice (Figure 2G). We also observed similar findings with another liver fibrosis model driven by choline-deficient L-amino-defined (CDAA) diet. We fed this diet to FDG mice for 8 weeks and injected DT for the final two weeks to examine liver fibrosis (Supplementary Figure 1A). Similar to the CCl_4_ model, Treg cells significantly were increased in the liver by feeding the diet (Supplementary Figures 1B,C) and Treg depletion aggravated liver fibrosis assessed by liver fibrotic regions and expression levels of *Col1a1* and *Acta2* (α-SMA) (Supplementary Figures 1D,E). Taken together, these data indicated that hepatic Treg cells predominantly proliferate in response to CCl_4_-induced liver injury and suppress chronic inflammation as well as the progression of liver fibrosis.

### Treg Cell-Derived Amphiregulin Is Dispensable for Preventing CCl_4_-Induced Liver Pathology

**FIGURE 3:**
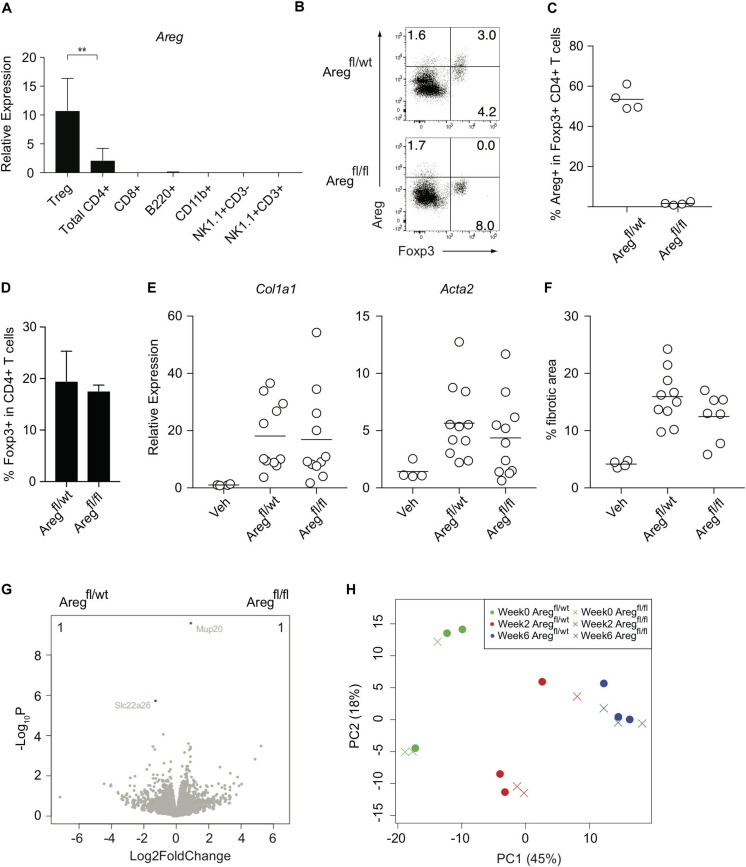
Treg-derived amphiregulin is dispensable for preventing CCl_4_-induced liver pathology. **(A)** Quantitative RT-PCR analysis of the amphiregulin gene *(Areg)* in CD4+ eGFP+ T (Treg) cells, CD4+ T cells, CD8+ T cells, B220+ B cells, CD11b+ myeloid cells, NK1.1+ CD3- NK cells, and NK1.1+CD3+ NKT cells isolated from the inflamed liver of FDG mice after the 6-week treatment with CCl_4_ (*n* = 2–7). **(B)** Intracellular Areg staining of hepatic CD4+ T cells from CCl_4_-treated Foxp3^YFP–Cre^ Areg^fl/wt^ or Foxp3^YFP–Cre^ Areg^fl/fl^ mice. **(C)** Proportions of Areg expression in hepatic Foxp3+CD4+ T cells as in panel **(B)** (*n* = 4 each). **(D)** Proportions of Foxp3 expression in hepatic CD4+ T cells in Foxp3^YFP–Cre^ Areg^fl/wt^ or Foxp3^YFP–Cre^ Areg^fl/fl^ mice. **(E)** Quantitative RT-PCR analysis for the expression of *Col1a1* and *Acta2* in the liver tissue after CCl_4_ treatment. **(F)** Proportions of fibrotic areas of the liver evaluated by Sirius red staining in CCl_4_-treated Foxp3^YFP–Cre^ Areg^fl/wt^ or Foxp3^YFP–Cre^ Areg^fl/fl^ mice or vehicle (Veh)-treated control mice. **(G)** RNA-seq analysis of whole liver tissues from Foxp3^YFP–Cre^ Areg^fl/wt^ or Foxp3^YFP–Cre^ Areg^fl/fl^ mice 6 weeks after CCl_4_ treatment. The volcano plots show genes with an adjusted *P* value < 0.05 in DESeq2 analysis between the groups. The genes upregulated in Foxp3^YFP–Cre^ Areg^fl/wt^ mice are shown in blue, and the genes downregulated are shown in red. The number of the genes is shown in the right and left top of the plots. **(H)** PCA analysis of liver RNA-seq data from the following six groups: Foxp3^YFP–Cre^ Areg^fl/wt^ or Foxp3^YFP–Cre^ Areg^fl/fl^ mice before CCl_4_ treatment (Week 0), or 2 weeks or 6 weeks after the treatment (Week 2) or (Week 6), respectively. Horizontal bars indicate the means in panels **(C,E,F)**. Vertical bars denote SD; **p < 0.01. Data are representative of two independent experiments in panel **(A)**. Data are pooled from two independent experiments in panels **(E,F)**.

We next investigated the Treg-mediated molecular mechanisms underlying the prevention of liver inflammation and fibrosis in this model. Amphiregulin produced by immune subsets such as Treg cells and innate lymphoid cells has recently been highlighted as a key molecule for tissue regeneration and repair during acute and chronic inflammation (9–11, 23, 24). Among the immune cells in the CCl_4_-treated inflamed liver, which included CD4+ and CD8+ T cells, B220+ B cells, CD11b+ myeloid cells, NK1.1+ NK cells, and NK1.1+CD3+NKT cells, the *Areg* gene encoding amphiregulin was predominantly expressed by Treg cells (Figure 3A). To determine the specific effect of Treg-derived amphiregulin on CCl_4_-induced liver pathology, we generated Foxp3^YFP–Cre^ Areg^fl/fl^ mice in which the expression of amphiregulin from Treg cells was selectively deleted (Figures 3B,C). The absence of amphiregulin in Treg cells did not affect the proportions of hepatic Treg cells as compared with control Foxp3^YFP–Cre^ Areg^fl/wt^ mice (Figure 3D). We then treated Foxp3^YFP–Cre^ Areg^fl/wt^ or Foxp3^YFP–Cre^ Areg^fl/fl^ mice with vehicle or CCl_4_, and assessed gene expression profiles by q-PCR and RNA-seq, and liver fibrosis by staining with Sirius red. Despite the critical role of amphiregulin in various inflammation models, there were no significant changes in the expression of *Col1al* and *Acta2* and Sirius red-positive fibrotic areas in the liver of Foxp3^YFP–Cre^ Areg^fl/wt^ or Foxp3^YFP–Cre^ Areg^fl/fl^ mice (Figures 3E,F). RNA-seq analysis of liver tissues from Foxp3^YFP–Cre^ Areg^fl/wt^ or Foxp3^YFP–Cre^ Areg^fl/fl^ mice six weeks after CCl_4_ treatment further revealed that the gene profiles between the two groups were almost identical with two minor genes with adjusted P value less than 0.05 in DESeq2 analysis. (Figure 3G). In addition, we collected liver tissue samples from Foxp3^YFP–Cre^ Areg^fl/wt^ or Foxp3^YFP–Cre^ Areg^fl/fl^ mice for RNA-seq analysis at three different time points before or during the course of CCl_4_ treatment (weeks 0, 2, and 6) and performed principal component analysis. No remarkable difference in the gene expression profiles, including markers for tissue fibrosis and T cell-mediated inflammation, were observed at any time point (Figure 3H and Table 1). These results demonstrated that the Treg-derived amphiregulin is dispensable for maintaining liver homeostasis and preventing CCl_4_-induced liver pathology.

**TABLE 1:** Differentially expressed genes between Foxp3^*YFP*–*Cre*^ Areg^*fl*/*wt*^ and Foxp3^*YFP*–*Cre*^ Areg^*fl*/*fl*^ mice at the three time points.

**Week 0**				
**Gene_Symbol**	**Base mean**	**log2 fold change**	***p* value**	**padj**
**Upregulated in Areg^*fl*/*wt*^**				
Serpina7	416.4	1.3	6.87E-08	2.45E-04
Lpar2	51.4	1.3	1.21E-05	1.84E-02
**Upregulated in Areg^fl/fl^**				
Gadd45g	502.4	1.4	9.08E-07	2.43E-03

**Week 2**				
**Gene_Symbol**	**Base mean**	**log2 fold change**	***p* value**	**padj**

**Upregulated in Areg^fl/wt^**				
Hcn3	344.0	1.3	3.15E-06	4.95E-03
Gadd45b	101.8	1.3	5.51E-05	3.14E-02
Rdh18-ps	71.9	1.3	1.36E-05	1.31E-02
Ccrn4l	718.7	1.2	8.60E-06	9.83E-03
Sik1	687.7	1.1	4.07E-07	1.02E-03
Plk3	1235.4	1.1	1.94E-06	3.48E-03
**Upregulated in Areg^fl/fl^**				
Cyp8b1	1533.7	1.3	2.93E-07	9.23E-04
Ccdc120	87.4	1.1	5.28E-05	3.14E-02
Nab2	455.7	1.1	1.15E-11	1.45E-07

**Week 6**				
**Gene_Symbol**	**Base mean**	**log2 fold change**	***p* value**	**padj**

**Upregulated in Areg^fl/wt^**				
Slc22a26	847.8	1.2	1.88E-06	1.68E-02
**Upregulated in Areg^fl/fl^**				
None				

*A gene list from RNA-seq analysis of whole liver tissues between Foxp3^*YFP–Cre*^ Areg^*fl/fl*^ (Areg^*fl/fl*^) and Foxp3^*YFP–Cre*^ Areg^*fl/wt*^ (Areg^*fl/wt*^) mice at Week 0, Week 2, and Week 6 after CCl_4_ treatments as defined in section “Materials and Methods.” Genes with adjusted *p* value < 0.05 and log2 fold change > 1 were shown.*

### Hepatic Treg Cells Suppress Inflammatory Cellular Immunity Associated With Tissue Fibrosis

**FIGURE 4:**
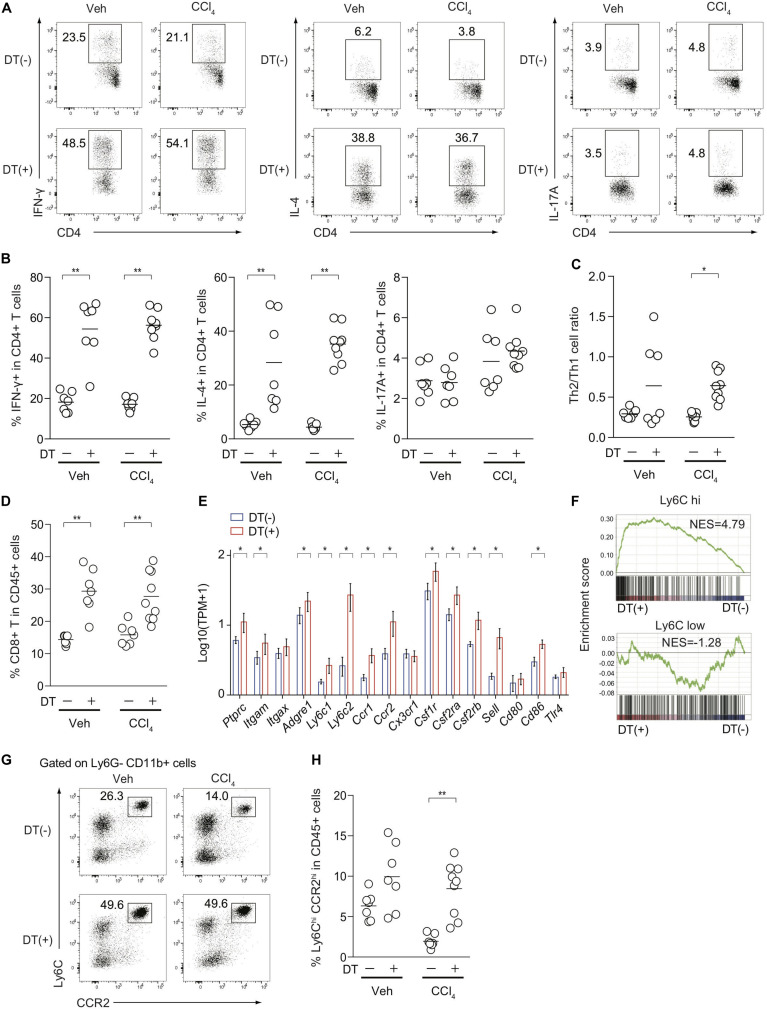
Treg-cell depletion in CCl_4_-treated FDG mice induces the activation and expansion of pro-fibrotic cellular immunity. **(A,B)** Proportions of IFN-γ, IL-4, and IL-17-producing hepatic CD4+ T cells from DT(−) or DT(+) FDG mice after vehicle (Veh) or CCl_4_ treatment. **(C)** Th2/Th1 cell ratios of hepatic CD4+ T cells from DT(−) or DT(+) FDG mice after Veh or CCl_4_ treatment. **(D)** Proportions of CD8a+ T cells in hepatic CD45.2+ cells after treatment. **(E)** Gene expression profiles of hepatic monocyte/macrophage associated genes from RNA-seq data of CCl_4_-treated DT(−) or DT(+) FDG mice (*n* = 3–4). Vertical bars denote SD. **(F)** Gene set enrichment analysis of liver RNA-seq data from DT(−) and DT(+) FDG mice with Ly-6C^*high*^- and Ly-6C^*low*^-enriched signature as detailed in section “Materials and Methods.” **(G,H)** Proportions of Ly-6C^*high*^ CCR2^*high*^ cells in hepatic Ly-6G- CD11b+ cells **(G)** and CD45.2+ cells **(H)** from CCl_4_-treated DT(−) or DT(+) FDG mice. Horizontal bars indicate the means in panels **(B–D,H)**; *p < 0.05, **p < 0.01. Data are representative of two independent experiments in panels **(A,G)**. Data are pooled from two experiments in panels **(B–D,H)**.

Because Treg depletion exacerbated CCl_4_-induced liver damage and fibrosis (Figure 2), we assessed inflammatory cellular components in the inflamed liver from FDG mice with or without DT treatment. Although it is technically challenging to detect small numbers of antigen-specific immune responses induced by CCl_4_-mediated liver damages, the proportions of hepatic Th1 and Th2 cells, but not Th17 cells, in both vehicle- and CCl_4_-treated FDG mice, were dramatically increased upon DT administration (Figures 4A,B). The mediators involved in fibrogenesis are distinct from those involved in inflammation (25). In contrast to the type 2 cytokine IL-4 signaling as a pro-fibrotic mediator, IFN-γ has been characterized as an anti-fibrotic cytokine in the context of CCl_4_-mediated liver fibrosis (26, 27). Notably, the Th2/Th1 cell ratio, which is associated with tissue fibrosis, was specifically increased by the combination of CCl_4_ treatment and Treg depletion, but not by Treg depletion alone (Figure 4C). The proportions of CD8+ cytotoxic T cells in the CD45+ population also increased (Figure 4D). We next focused on Ly-6C^high^ inflammatory monocytes/macrophages, which are also reported as a pro-fibrotic immune subset (20, 28), and compared the expression of relevant genes in the inflamed liver of CCl_4_-treated FDG mice with or without DT. Several cell surface markers for Ly-6C^high^ inflammatory monocytes/macrophages, such as *Ly6c2*, *Ccr2*, *Csf1r*, and *Sell*, were significantly upregulated in Treg-depleted FDG mice (Figure 4E). In addition, GSEA revealed the significant enrichment of Ly-6C^high^ inflammatory monocytes/macrophage signatures by Treg depletion. Ly-6C^low^ macrophage signatures, which are supposed to be an anti-fibrotic subset, were unchanged (Figure 4F). We also validated this finding by FACS analysis. The frequencies of Ly-6C^high^ CCR2^high^ inflammatory monocytes/macrophages were substantially expanded in the Treg-depleted fibrotic liver (Figures 4G,H). Collectively, these data indicated that Treg cells suppress the aberrant activation and expansion of the pro-fibrotic cellular immunity, especially Th2 cells and Ly-6C^high^ CCR2^high^ inflammatory monocytes/macrophages, during CCl_4_-induced liver inflammation, thereby inhibiting the progression of liver fibrosis.

## Discussion

The current research focus seeks to understand tissue adaptation and organ-specific roles of Treg cells under physiological and pathological conditions. Accumulated evidence has indicated diverse immune suppression mechanisms of tissue Treg cells that operate in a cell-contact or secretory molecule-dependent manner (3). Moreover, recent findings have provided novel insight into Treg non-canonical functions in tissue regeneration and repair by the crosstalk between Treg and mesenchymal cells through amphiregulin in the inflamed brain, muscle, and lung (9–11). In this study, we demonstrate that Treg cells prevent liver pathology in a CCl_4_-induced liver fibrosis model by limiting the aberrant activation of pro-fibrotic cellular immunity, including Th2 cells and Ly-6C^high^ CCR2^high^ inflammatory monocytes/macrophages. Conversely, the tissue protective role of Treg-derived amphiregulin was limited in CCl_4_-induced liver inflammation and fibrosis, despite the predominant expression of amphiregulin in hepatic Treg cells.

Hepatic Treg cells sense various inflammatory cues to proliferate (29, 30) and regulate the pathogenesis of liver fibrosis in an acute disease model induced by bile duct ligation (29, 31). In addition, Foxp3+ human Treg cells expand in the fibrotic liver of hepatitis C virus infected patients (7). Although it was suggested that Treg cells might promote liver fibrosis based on Treg depletion experiments by anti-CD25 mAbs, the treatment of the antibody could also deplete pro-fibrotic effector T cells simultaneously, making the interpretation of the effect of the antibody on Treg or effector T cells difficult (8). In this study using FDG mice in which Foxp3+ Treg cells can specifically be depleted, we demonstrated that CCl_4_-induced liver inflammation and fibrosis also drive hepatic Foxp3+ Treg expansion, suggesting that common self-antigens or damage-associated molecular patterns (DAMPs) including IL-33 released from stressed or damaged hepatocytes are likely to stimulate Treg cells to proliferate and regulate excessive inflammation and liver pathology. Treg cells possess TCRs with high self-reactivity, which are key characteristics for mediating immunological self-tolerance (32, 33). Conversely, the experimental removal of Treg cells or Foxp3 mutations in mice and humans causes systemic autoimmune diseases, including liver inflammation (12, 34, 35). Thus, self-reactive Treg cells in the liver actively engage in liver homeostasis and prevent liver pathology resulting from hepatotoxic chemicals and immune activation. We found that Treg cells preferentially regulate cellular signatures during chronic liver inflammation, such as Th2 cells and Ly-6C^high^ CCR2^high^ inflammatory monocytes/macrophages, which were previously reported to produce the pro-fibrotic mediators IL-4 and TGF-β, respectively (28, 36, 37) and may be regulated by Treg-derived IL-10 (38). Group 2 innate lymphoid cells are also considered a pro-fibrotic population in the liver and promote liver fibrosis in an IL-33 dependent manner in the CCl_4_ model (39). These pro-fibrotic mediators activate hepatic stellate cells to secrete extracellular matrix proteins for the progression of liver fibrosis (40). However, it remains to be determined whether hepatic Treg cells directly control cellular immunity and stellate cells *in situ*, or whether Treg cells located in lymphoid organs like lymph nodes suppress the activation and trafficking of the effector cells before infiltrating into the liver.

Tissue Treg cells are capable of adapting to organ-specific environmental cues, including DAMPs, and subsequently acquire specific functions to control immune responses and tissue repair. In damaged tissue, IL-33, a well-known alarmin or DAMP, is abundantly released from necrotic cells and stimulates local Treg cells via signals from ST2, an IL-33 receptor, to produce amphiregulin, an epithelial growth factor receptor ligand (10). Recently, cell type-specific amphiregulin has been highlighted as a key molecule for tissue regeneration and repair in a context-dependent manner (9–11). Amphiregulin can also mediate protection of injured liver tissue by promoting hepatocyte proliferation (41, 42). Our RNA-seq data using Foxp3^YFP–Cre^ Areg^fl/fl^ mice clearly indicated that Treg-derived amphiregulin is not required to maintain liver homeostasis or to protect from liver inflammation and fibrosis through amphiregulin-mediated survival signals and cell growth, at least in CCl_4_-induced chronic liver injury, although Treg cells predominantly produce amphiregulin among immune cells in the liver. However, this finding does not necessarily exclude the possibility that hepatic Treg cells play a protective role in directly modulating regeneration of injured hepatocytes through unappreciated mechanisms. Further work is needed to explore this aspect of hepatic Treg non-canonical functions that can act on mesenchymal cells to prevent liver pathology during chronic inflammation.

## Data Availability Statement

The datasets presented in this study can be found in online repositories. The names of the repository/repositories and accession number(s) can be found below: https://ddbj.nig.ac.jp/DRASearch/submission?acc=DRA010411.

## Ethics Statement

The animal study was reviewed and approved by the Ethical Committee of Institute for Frontier Life and Medical Sciences and Graduate School of Medicine, Kyoto University.

## Author Contributions

YI, DO, and KH designed the study. YI, DO, YT, HW, GK, and KH performed the experiments. DO performed the statistical analysis and bioinformatics analysis of the RNA-seq data. KT and SU provided intellectual input. YI, DO, YT, and KH wrote the manuscript. KH supervised the project. All authors reviewed the manuscript.

## Conflict of Interest

The authors declare that the research was conducted in the absence of any commercial or financial relationships that could be construed as a potential conflict of interest.
