# Supplementary material for: Foxp3+ Regulatory T Cells Inhibit CCl4-Induced Liver Inflammation and Fibrosis by Regulating Tissue Cellular Immunity
Source: Front Immunol. 2020 Oct 15;11:584048. doi: 10.3389/fimmu.2020.584048 (PMC7593684; doi:10.3389/fimmu.2020.584048)
Supplement: Supplementary file 1 [file Figure_1.DOCX]

Supplementary Material

**Foxp3+ regulatory T cells inhibit CCl_4_-induced liver inflammation and fibrosis by regulating tissue cellular immunity**

Yoshinobu Ikeno, Daiya Ohara, Yusuke Takeuchi, Hitomi Watanabe, Gen Kondoh, Kojiro Taura, Shinji Uemoto, Keiji Hirota

.

## Supplementary Figure 1

##
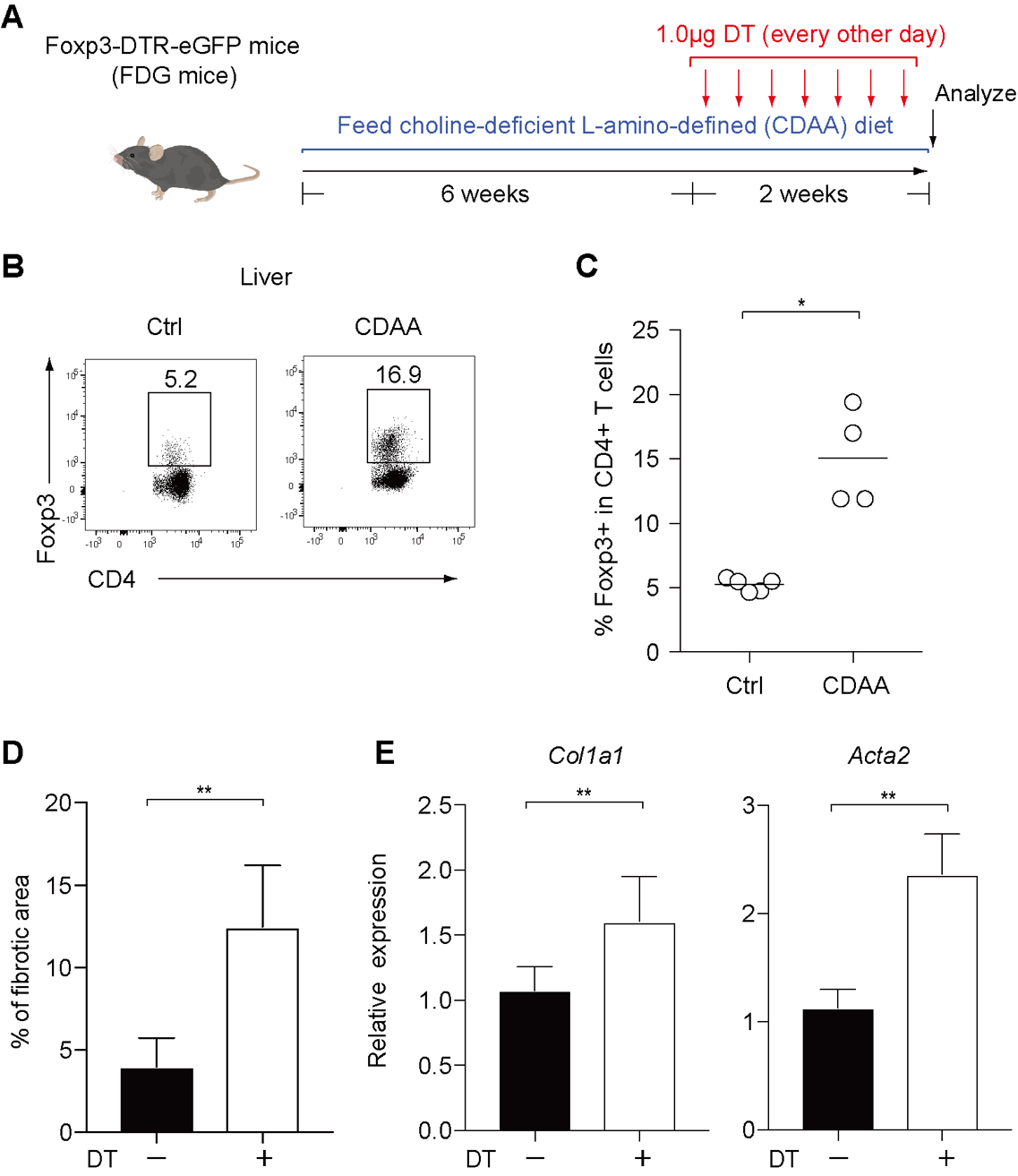


## Supplementary Figure 1. Treg-cell depletion aggravated liver fibrosis in chorine-deficient L-amino-defined (CDAA) diet model.

**(A)** Experimental design of CDAA diet model. Liver fibrosis was induced by feeding CDAA diet for 8 weeks. For Treg-cell depletion, FDG mice were untreated or administered diphtheria toxin (DT) during the final two weeks of CDAA diet treatment. **(B-C)** Proportions of Foxp3+ in hepatic CD4+ T cells of mice fed with normal (Ctrl) or CDAA diet (n=4~5). **(D)** Quantification of fibrotic areas of the liver sections evaluated by Sirius red positive areas (n>5). **(E)** Quantitative RT-PCR analysis for the expression of *Col1al* and *Acta2* (α-SMA) in the liver tissues of DT(-) and DT(+) FDG mice fed with CDAA diet (n>4). Horizontal bars in **C** indicate the means. Vertical bars denote SD; *p<0.05, **p<0.01. Data are representative of two independent experiments in **B** and **C**. Data are pooled from two experiments in **D** and **E**.
